# Assessing the Value of Prehabilitation in Patients Undergoing Colorectal Surgery According to the Enhanced Recovery After Surgery (ERAS) Pathway for the Improvement of Postoperative Outcomes: Protocol for a Randomized Controlled Trial

**DOI:** 10.2196/resprot.7972

**Published:** 2017-10-27

**Authors:** Cornelia Merki-Künzli, Marta Kerstan-Huber, Denise Switalla, David Gisi, Dimitri Aristotle Raptis, Nicola Greco, Giuseppe Mungo, Markus Wirz, Severin Gloor, Merima Misirlic, Stefan Breitenstein, Christoph Tschuor

**Affiliations:** ^1^ Department of Surgery Cantonal Hospital Winterthur Winterthur Switzerland; ^2^ Institute of Physiotherapy Cantonal Hospital Winterthur Winterthur Switzerland; ^3^ Division of Visceral Surgery and Transplantation University Hospital Zurich Zurich Switzerland; ^4^ Institute of Physiotherapy Zurich University of Applied Sciences Winterthur Switzerland

**Keywords:** prehabilitation, enhanced recovery after surgery, ERAS

## Abstract

**Background:**

A key element in the postoperative phase of the standardized Enhanced Recovery After Surgery (ERAS) treatment pathways is mobilization. Currently, there are no recommendations in the ERAS guidelines for preoperative physical activity. Patients undergoing major surgery are prone to functional decline due to the impairment of muscle, cardiorespiratory, and neurological function as a response to surgical stress. It has been shown that preoperative physical training reduces postoperative complications. To date, there are limited studies that investigate preoperative physical training combined with ERAS.

**Objective:**

The aim of this study is to assess the impact of tailored physical training prior to colorectal surgery conducted according to an ERAS protocol on overall morbidity. This study proposes the initial hypothesis that 3-6 weeks of prehabilitation before elective colorectal surgery may improve postoperative outcome and reduce complication rates, assessed using the Comprehensive Complication Index. The primary objective is to evaluate overall morbidity due to postoperative complications. Additionally, complications are assessed according to the Clavien-Dindo classification, length of stay, readmission rate, mortality rate, and treatment-related costs.

**Methods:**

The prehabilitation Enhanced Recovery After colorectal Surgery (pERACS) study is a single-center, single-blinded prospective randomized controlled trial. Patients scheduled for colorectal resections are randomly assigned either to the prehabilitation group or the control group. All patients are treated with the ERAS pathway for colorectal resections according to a standardized study schedule. Sample size calculation performed by estimating a clinically relevant 25% reduction of postoperative complications (alpha=.05, power 80%, dropout rate of 10%) resulted in 56 randomized patients per group.

**Results:**

Following ethical approval of the study protocol, the first patient was included in June 2016. At this time, a total of 40 patients have been included; 27 patients terminated the study by the end of March 2017. Results are expected to be published in 2018.

**Conclusions:**

The pERACS trial is a single-center, single-blinded prospective randomized controlled trial to assess the impact of tailored physical training prior to colorectal surgery, conducted according to an ERAS protocol, in order to evaluate overall morbidity.

**Trial Registration:**

Clinicaltrials.gov NCT02746731; https://clinicaltrials.gov/ct2/show/NCT02746731 (Archived by WebCite at http://www.webcitation.org/6tzblGwge)

## Introduction

During the 1990s, Henrik Kehlet, a Danish surgeon, developed the concept of fast-track surgery [1]. By optimizing perioperative care, recovery was accelerated. To advance this, the Enhanced Recovery After Surgery (ERAS) concept was developed by Scandinavian and British surgeons. ERAS is an evidence-based multimodal treatment concept to improve postoperative recovery. In contrast to the fast-track concept, the ERAS guidelines do not only focus on pace. Instead, the multifaceted ERAS approach aims to reduce perioperative stress, improve postoperative recovery, reduce morbidity and mortality rates, and shorten the length of hospital stay (LOS). The first ERAS guidelines for elective colorectal surgery were published in 2005 [2].

In the postoperative phase, one of the most important elements pertaining to physical activity is mobilization. According to the ERAS approach, the goal is to get patients out of bed on the same day as the surgery, for at least 4 hours on postoperative day (POD) 1 and for at least 6 hours from POD 2 onwards. However, there are no recommendations for physical activity prior to surgery.

Patients undergoing major surgery are prone to physical decline due to preexisting reduced general health and the impairment of muscle, cardiorespiratory, and neurological function in response to surgical stress. While physically healthy patients have the capacity to cope with this stress response, patients with poorer preoperative physical conditions might not have this capacity. Therefore, these patients are at a higher risk for postoperative complications [3].

In elective cardiac surgery, there is evidence that preoperative physiotherapy reduces postoperative pulmonary complications [4]. Similar effects are suggested for thoracic, abdominal, and major joint replacement surgery [3]. Dronkers at al reported an association between preoperative physical fitness/physical activity and outcome after scheduled major abdominal surgery [5].

To date, there have been only a few studies investigating preoperative physical training combined with ERAS [6]. In respect to existing evidence, which suggests potential benefits through preoperative physical training, one objective of this study is to investigate these findings in the setting of an ERAS approach for colorectal resections. The primary aim of the study is to examine whether moderate to intense physical training (partially supervised by a qualified person), implemented within a short timeframe prior to surgery, will reduce overall morbidity and mortality rates in the ERAS cohort. The hypothesis of the trial is that 3-6 weeks of prehabilitation before elective colorectal surgery improves postoperative outcome.

## Methods

### Study Design

The prehabilitation Enhanced Recovery After Colorectal Surgery (pERACS) trial is a single-center, single-blinded prospective randomized controlled trial to assess the impact of tailored physical training on overall morbidity, prior to colorectal surgery, conducted according to an ERAS approach. Patients with an indication for elective colorectal resection will be randomized into either the intervention group, with preoperative physical training, or the control group without preoperative physical training. Apart from the physiotherapeutic mobilization according to the protocol and ERAS guidelines, all patients will undergo their normal physical activities. All patients will be treated along the ERAS pathway for colorectal resections.

### Patients and Setting

Patients with an indication for elective colorectal resection will be eligible for this study, which will be carried out in the Department of Surgery of the Cantonal Hospital in Winterthur, Switzerland. Patients will be recruited for the study by senior surgeons who will also perform the surgical intervention.

#### Inclusion Criteria

Patients will be included based on the following criteria: adult patients over age 18 with or without comorbidities (eg, diabetes, obesity, cardiovascular disease), patients suffering from colorectal diseases (eg, colorectal cancer, diverticulosis, benign tumors such as polyps or inflammatory bowel disease), patients needing an operative treatment (eg, rectosigmoid resection, anterior resection of rectum, ileocecal/right hemicolectomy, left hemicolectomy, abdominoperineal resection, or total/subtotal colectomy), patients treated according to ERAS, patients undergoing reversal of stoma and Hartmann procedures treated according to ERAS, and informed consent as documented by signature.

#### Exclusion Criteria

Patients will be excluded if they are under age 18, are not able to provide informed consent, have a physical impairment (eg, paresis) or severe cardiac or pulmonary comorbidities (NYHA III or IV), cannot perform the necessary physical training, are not able or willing to attend the physical training at the institute of physiotherapy, are not able to follow the procedures of the study (eg, due to language restrictions, psychological disorders), are participating in another study with investigational drugs 30 days preceding and during the study, or enrollment of the investigator, his/her family members, employees, and other dependent persons.

### Sample Size Calculation

The sample size calculation was performed assuming a clinically relevant 25% reduction of postoperative complications as assessed by the Comprehensive Complication Index (CCI) favoring the treatment group (prehabilitation) when compared to the control group (no prehabilitation) [7,8]. Using a dataset of 47 patients who underwent ERAS colorectal surgery in our department, we determined the mean CCI was 10 (SD 5). Thus, the treatment group mean was set at 7.5, and the standard deviation was adjusted accordingly (SD 3.75) [9]. The alpha error was typically set at .05 and power at 80%. The initial total sample size of 102 patients was increased by 10% to adjust for potential of loss to follow-up resulting in a total sample size of 112 participants (ie, 56 participants per group respectively).

### Intervention

Patients in the intervention group will train for 3-6 weeks prior to surgery, twice a week at the institute of physiotherapy under the guidance of a qualified physiotherapist and once unsupervised at home. Training will be tailored and constantly adapted according to the actual condition of the patient. The program comprises both strengthening and endurance components (see Table 1). In addition, patients will be informed about the importance of their physical condition with respect to the postoperative course and will be encouraged to adhere to the training program, as well as to remain as physically active as possible in addition to the physical exercise training program. Each supervised training session will last 90 minutes and will include the following elements:

- Warm-up: guided movements of all main articulations in order to prevent injuries and increasing activity in order to prepare the cardiovascular system for exercise
- Aerobic interval training on a bicycle: high-intensity interval training with a duration of 32 minutes (4x4mins with 85-90% of maximum training capacity)
- Resistance training: circuit training on six different devices allowing the strengthening of large muscle groups of the arms and legs
- Cool-down: guided stretching of the previously trained muscles

Patients assigned to the control group receive instructions about the importance of their physical condition with respect to the postoperative course and are encouraged to remain physically active. This is in accordance with the current standard procedure. All patients will be asked to record their physical activity in a diary. Regardless of the group allocation, all patients will be treated according to the ERAS approach.

#### The Steep Ramp Test

The steep ramp test is a validated short maximal exercise capacity test that does not require respiratory gas analysis measurements and has been described in the exercise rehabilitation of patients with chronic heart failure. The main outcome of the steep ramp test is the achieved work rate peak, which partially reflects anaerobic power and leg muscle strength [10]. The test has been proved to be safe, reproducible, and practical in use for prescribing the training load and for monitoring training progress in the rehabilitation of cancer patients [11].

#### 2-Minute Walk Test

The 2-Minute Walk Test is a measurement of endurance by assessing walking distance over 2 minutes. It is a simple test that can be widely used in clinical practice as well as for research [12].

#### Five Times Sit to Stand Test

The Five Times Sit to Stand Test (FTSST) is a quick and easy test to perform. It measures the time (in seconds) that an individual needs to change between sitting (standard chair with arms and a seat height of 43 cm) and standing five times in a row. FTSST is a multidimensional task that is associated with lower extremity strength and balance.

#### Hand Grip Strength

Reduced grip strength has been shown to be a predictor of impaired short-term outcome, such as increased postoperative complications, increased length of hospitalization, higher readmission rate, and decreased physical status [13]. For the grip strength, the average value of three successive measurements of the dominant hand with a Jamar dynamometer [14,15] will be calculated.

#### Fatigue-Visual Analogue Scale

The Fatigue-Visual Analogue Scale (F-VAS) is a horizontal line, 100 mm in length, anchored by word descriptors at each end. Patient mark on the line the point representing their perception of their fatigue. The F-VAS score is determined by measuring in millimeters from the left end of the line to the point that the patient marks.

#### Pain (Numeric Rating Scale)

Level of pain will be assessed by asking patients prior to and after the physiotherapy session to rate their perceived pain intensity using a numeric rating scale (NRS). The NRS is an 11-point scale from 0-10, where “0” indicates no pain and “10” indicates the maximum pain imaginable [16].

#### International Physical Activity Questionnaire ‒ Short Form

The International Physical Activity Questionnaire ‒ short form (IPAQ-SF) will be used in this study because the ease of administration (ie, the burden on participants to report their activity) is small [17]. The IPAQ-SF (9 items) records the activity of four intensity levels: (1) vigorous-intensity activity such as aerobics, (2) moderate-intensity activity such as leisure cycling, (3) walking, and (4) sitting.

#### European Organisation for Research and Treatment of Cancer Quality of Life Questionnaire C30

The European Organisation for Research and Treatment of Cancer Quality of Life Questionnaire C30 is a questionnaire developed to assess the quality of life of cancer patients. It is an instrument that has been translated and validated in over 90 languages and is used in more than 3000 studies worldwide.

**Table 1:** Administration of study intervention and control intervention.

	Body structure and function	Activity	Questionnaires and other tests
Preoperative (3-6 weeks before surgical intervention)	Steep ramp test	Five Times Sit to Stand	International Physical Activity Questionnaire ‒ short form	
	Fatigue-Visual Analogue Scale	2-Minute Walk Test	European Organisation for Research and Treatment of Cancer Quality of Life Questionnaire C30
	Pain Numeric Rating Scale		
	Hand grip strength		
Surgical intervention	Fatigue-Visual Analogue Scale		Adherence to exercise	
	Pain Numeric Rating Scale			
	Pulse and oxygenation			
Postoperative during hospitalization	Fatigue-Visual Analogue Scale	Five Times Sit to Stand	European Organisation for Research and Treatment of Cancer Quality of Life Questionnaire C30
	Pain Numeric Rating Scale	2-Minute Walk Test	
	Hand grip strength	Modified Iowa Levels of Assistance Scale	
Postoperative (6 weeks after surgical intervention)	Steep ramp test	Five Times Sit to Stand	International Physical Activity Questionnaire ‒ short form	
	Fatigue-Visual Analogue Scale	2-Minute Walk Test	European Organisation for Research and Treatment of Cancer Quality of Life Questionnaire C30
	Pain Numeric Rating Scale		
	Hand grip strength		

#### Assessment of Postoperative Functional Recovery During Hospital Stay: Modified Iowa Levels of Assistance Scale

For assessment of postoperative functional recovery, the modified version of the Iowa Levels of Assistance Scale (MILAS) [18] will be obtained daily. The MILAS assesses the ability of patients to safely perform four activities of daily life (supine to sit, sit to stand, walking, and stair climbing) and rates the amount of assistance needed. This measurement enables the physiotherapist to assess whether and when a patient can function independently and allows tailoring treatment goals.

### Endpoints

As the primary endpoint, the complication rate will be assessed in-hospital and at the 30-day follow-up using the CCI [19,20]. Secondary endpoints are the complications assessed according to Clavien/Dindo [21], LOS, readmission rate, mortality rate, and costs as well as measures associated with physical performance. To compare the two groups and follow the progress in the intervention group, tests and questionnaires will be used (see Table 2).

### Study Procedures

Patients are allocated to our consultation center by general practitioners or gastroenterologists. They will be recruited to the study during normal consultation through senior surgeons. We will include all consecutive patients electively treated for colorectal diseases in accordance with the ERAS approach, for colorectal resections from June 13, 2016, onwards. Patients will be randomly assigned either to the control or to the intervention group after entering their data into the Web-based database. All complications will be recorded and assessed according to the Clavien-Dindo classification at the time of discharge and follow-up, by the unblinded study nurse (Table 2). Results will be transformed to the CCI score. The LOS and readmission rate will be recorded at the time of discharge and follow-up. Cost analysis will be performed with the local department of finance.

### Analyses

The statistical analysis will be performed on an intention-to-treat basis by the study’s independent statistician. For each patient, basic demographic, prehabilitation, intraoperative, postoperative, and follow-up data will be generated and stored in a password-protected and encrypted database. These data will be compared separately for each randomization group. Both significant as well as nonsignificant results will be reported accordingly.

The primary endpoint (CCI) will be compared between the two randomized groups (prehabilitation/treatment group vs no prehabilitation/control group) using the Student *t* test. The CCI is known from other trials on postoperative complications to be normally distributed [19,20].

As for the secondary outcomes, the two randomized groups will be compared with the Pearson chi-square test with regards to the Clavien/Dindo classification [21]. This is typically an ordinal variable. Furthermore, the LOS, typically with a skewed distribution, will be compared between the two randomized groups with the Mann-Whitney U test. Readmission and mortality rates will be compared with the Fisher’s exact test between the two groups. The overall total in-hospital costs between the two randomized groups will be compared either with the Student *t* or the Mann-Whitney U-test, depending on their normality of distribution. Cost-effectiveness between the two groups will be compared. All *P* values will be 2-sided and considered statistically significant, if *P*<.05. The statistical analysis will be performed on SPSS 22 for Mac.

A subgroup analysis to investigate whether disease entity (benign vs malignant disease) influences the impact of prehabilitation on the outcome will be performed. Due to the small sample size, further subgroup analysis (eg, for different procedures) will not be performed. There are no multivariable analyses planned for this study due to the nature of the study design (randomized controlled trial) as there will be no need for adjustment of the results.

An interim analysis will be performed as soon as the total sample size of the study reaches 56 patients for both groups. The purpose of the interim analysis is to assess whether the inclusion rate of patients is acceptable and as expected. Further, we analyze the possibility of unexpected rates of severe or life-threatening adverse events or the extraordinary favorable effectiveness of the intervention, which may indicate the premature closure of the trial. An ad hoc interim analysis will be performed after inclusion of 20 patients to assess practicability only.

**Table 2:** Physical tests and questionnaires.

	Intervention group		Control group	
	Preoperative	Postoperative	Preoperative	Postoperative
Steep Ramp Test	x	x	x	x
Borg Rating of Perceived Exertion Scale	x	x	x	x
Numeric Rating Scale Pain	x	x	x	x
Hand grip strength	x	x	x	x
Five Times Sit to Stand test	x	x	x	x
2-Minute Walk Test	x	x	x	x
International Physical Activity Questionnaire-short form	x	x	x	x
Modified Iowa Levels of Assistance		x		x
Comprehensive Complication Index		x		x
Clavien/Dindo Score		x		x
Length of stay		x		x
Readmission		x		x
Mortality rate		x		x
Costs		x		x

## Results

Following ethical approval of the study protocol, the first patient was included in June 2016; 40 patients have now been included, and 27 patients had terminated the study by the end of March 2017. Results are expected to be published in 2018.

## Discussion

### Principal Considerations

Since the introduction of the evidence-based ERAS treatment pathway, the rate of complications has been markedly reduced [22-24]. Equal observations were made in the study center (ie, Cantonal Hospital Winterthur unpublished data). A defining characteristic of ERAS is its multimodal, multidisciplinary approach. One component of ERAS is physical activity, more specifically the early postoperative mobilization of patients. With our study, we aim to investigate the effectiveness of physical training prior to the operation. There is an indication from other areas that physical preconditioning improves the resistibility of patients. The consequence is better toleration of operation-associated stress. However, these findings need to be confirmed for patients with colorectal disorders requiring surgical intervention according to an ERAS approach.

### Strengths and Limitations

An arbitrary choice in this study was the duration of the training and the number of training sessions. Practical aspects, the tolerated period of waiting time, and the risk for undesired progression of the underlying disease were taken into consideration as we defined a training period of 3-6 weeks. This was a consensus based on time needed to see any physical improvements and previous studies investigating the impact of treatment delay on outcome. To our best knowledge, there is no study showing that a delay of 3-6 weeks has an impact on the oncological outcome [25,26].

We believe that the strength of this study is the consideration of the whole treatment process as opposed to a single element. In addition the study can be considered a pragmatic trial since it represents daily clinical routine.

### Conclusion

The pERACS trial is a single-center, single-blinded prospective randomized controlled trial to assess the impact of tailored physical training prior to colorectal surgery, conducted according to an ERAS protocol, in order to evaluate overall morbidity.

## Abbreviations

CCI: Comprehensive Complication Index
ERAS: Enhanced Recovery After Surgery
FTSST: Five Times Sit to Stand Test
F-VAS: Fatigue-Visual Analogue Scale
IPAQ-SF: International Physical Activity Questionnaire short-form
LOS: length of hospital stay
MILAS: Modified Iowa Levels of Assistance Scale
NRS: Numeric Rating Scale
pERACS: Prehabilitation Enhanced Recovery After Colorectal Surgery
POD: postoperative day
